# Influence of Rhamnolipids and Ionic Cross-Linking Conditions on the Mechanical Properties of Alginate Hydrogels as a Model Bacterial Biofilm

**DOI:** 10.3390/ijms22136840

**Published:** 2021-06-25

**Authors:** Natalia Czaplicka, Szymon Mania, Donata Konopacka-Łyskawa

**Affiliations:** 1 Department of Process Engineering and Chemical Technology, Faculty of Chemistry, Gdańsk University of Technology, 80-233 Gdańsk, Poland; donkonop@pg.edu.pl; 2 Department of Chemistry, Technology and Biotechnology of Food, Faculty of Chemistry, Gdańsk University of Technology, 80-233 Gdańsk, Poland; szymon.mania@pg.edu.pl

**Keywords:** alginate hydrogel, rhamnolipids, ionic cross-linking, mechanical properties, biofilm

## Abstract

The literature indicates the existence of a relationship between rhamnolipids and bacterial biofilm, as well as the ability of selected bacteria to produce rhamnolipids and alginate. However, the influence of biosurfactant molecules on the mechanical properties of biofilms are still not fully understood. The aim of this research is to determine the effect of rhamnolipids concentration, CaCl_2_ concentration, and ionic cross-linking time on the mechanical properties of alginate hydrogels using a Box–Behnken design. The mechanical properties of cross-linked alginate hydrogels were characterized using a universal testing machine. It was assumed that the addition of rhamnolipids mainly affects the compression load, and the value of this parameter is lower for hydrogels produced with biosurfactant concentration below CMC than for hydrogels obtained in pure water. In contrast, the addition of rhamnolipids in an amount exceeding CMC causes an increase in compression load. In bacterial biofilms, the presence of rhamnolipid molecules does not exceed the CMC value, which may confirm the influence of this biosurfactant on the formation of the biofilm structure. Moreover, rhamnolipids interact with the hydrophobic part of the alginate copolymer chains, and then the hydrophilic groups of adsorbed biosurfactant molecules create additional calcium ion trapping sites.

## 1. Introduction

Alginates are naturally occurring polysaccharides [1]. They consist of β-D-mannuronic acid (M) and α-L-guluronic acid (G) residues, linked together by β-(1-4) glycosidic bonds [2,3]. The distribution of the M and G residues as well as the length of the polymer chain depend on the natural source and the method of alginate extraction [4]. The features that characterize alginates are biodegradability, high biocompatibility, and easiness of processing [5,6]. Due to the fact that alginates are hydrophilic polymers and have a high water absorption and retention ability, they can form hydrogels, which are three-dimensionally cross-linked networks with high water content [6,7]. The ionic or covalent cross-linking process is used to maintain the consistency of alginate hydrogels and prevent their dissolution in the aqueous phase [7]. In the case of the most common method, ionic cross-linking, divalent cations, such as Ca^2+^ or Ba^2+^, are used which interact with the carboxyl groups of the guluronic residues [6]. The cations diffuse into the sodium alginate solution, creating a gel characterized by a gradient of divalent ions. The degree of cross-linking of the polymer strongly influences the water absorption ability and the mechanical properties of the hydrogel [6]. Cross-linked alginate hydrogels find numerous applications in many fields, including waste removal agents [8,9], drug carriers [10,11], controlled drug release systems [12,13,14,15], wound dressing materials [16,17,18,19], food products [20,21], and tissue engineering [22,23,24]. Due to their high affinity for water and mechanical properties similar to soft tissue, they are also used as a material for the construction of scaffolds supporting and facilitating cell growth, multiplication, and differentiation [25].

Furthermore, alginate is one of the biofilm substances produced by bacteria [26], and alginate hydrogels are used as a model biofilm in laboratory research [27,28,29]. Real biofilms contain cells embedded in a matrix formed by extracellular polymeric substances (ECS) [28]. ECS fill the spaces between bacteria, provide a sticky framework to hold cells within a complex structure and produce mechanical cohesive stability [30]. Due to the complex and variable composition of real biofilms, research on the influence of various factors is often carried out with the use of model biofilms [27,28,29]. In our research, we focused on the influence of rhamnolipids on the mechanical properties of an alginate hydrogel. Rhamnolipids is a group of biosurfactants, which are produced by many microorganisms, including the most studied opportunistic pathogen *Pseudomonas aeruginosa* [31,32]. They are classified as glycolipids and consist of a mono- or disaccharide molecule connected by a glycosidic bond to a fatty acid. These biomolecules are surface active agents of biological origin due to their amphiphilic structure containing hydrophilic and hydrophobic groups [33,34]. Similarly to their synthetic counterparts (surfactants), due to their activity, they can participate in many processes taking place on the phase boundary [34]. Their properties include the ability to reduce surface and interfacial tension, as well as the ability to stabilize foams and emulsions, wetting ability, and antistatic effect [35,36]. In addition, biosurfactants play a significant role in the mobility and formation of biofilms by bacterial cells and increase the solubility and bioavailability of hydrophobic compounds. The literature indicates the existence of a relationship between rhamnolipids and bacterial biofilm. According to Davey et al. [37], rhamnolipids are responsible for maintaining the functionality of transport channels and affect the structure of the biofilm. It has also been found that the presence of rhamnolipids results in the exclusion of other strains from the biofilm structure [38]. Moreover, these biosurfactants induce the breakdown of bacterial biofilms and affect their structure during the early stages of formation and their subsequent maturation [39]. Thus, alginate hydrogels containing rhamnolipids were used in this research as a model matrix because alginate and rhamnolipids are an ingredient produced by *Pseudomonas* in biofilms.

There are few studies in the literature on the influence of surfactants on the structure of alginate hydrogels. Kaygusuz et al. [40] investigated the effect of addition of nonionic Brij 35 and anionic sodium dodecyl sulfate (SDS) surfactants on the mechanical properties of alginate hydrogels. It was shown that the use of a nonionic surfactant decreased the Young’s modulus, while the addition of an anionic agent increased the value of this parameter. This is due to the opposing effect of selected surfactants on the charge of the alginate matrix [40]. Stoppel et al. [41] conducted studies on the effect of surfactants (Pluronic^®^ F68 nonionic surfactant) at concentrations exceeding the CMC present in alginate hydrogel biomaterials on both their mechanical properties and the transport of biological molecules (riboflavin and BSA). With the addition of a surfactant, a change in dynamic viscosity and a decrease in mechanical strength were observed. Such studies have particular importance in optimizing the transport of biological molecules, especially proteins, when surfactant additives are included in hydrogel preparations for cellular applications and drugs [41].

However, the influence of rhamnolipid biosurfactants on the mechanical properties of alginate hydrogels has not been studied so far. Thus, the aim of this work is to determine the effect of rhamnolipids concentration, calcium chloride concentration, and ionic cross-linking time on the mechanical properties (compression load, flexibility, and cohesiveness) of alginate hydrogels using the Box–Behnken design (BBD) to construct an experimental plan.

## 2. Materials and Methods

### 2.1. Reagents

Sodium alginate (Sigma-Aldrich, viscosity of 1% wt. solution in water at 25 °C: 5–40 mPaꞏs, M/G ratio: 1.56), anhydrous calcium chloride (≥99.9%, POCH, Poland), commercial rhamnolipid biosurfactant (R90, 90% purity, AGAE Technologies LLC, Corvallis, OR, USA). All solutions were prepared using distilled water. Reagents were used without further purification.

### 2.2. CMC Determination

The critical micelle concentration (CMC) was determined by measuring the surface tension of the rhamnolipids containing aqueous solutions at room temperature (approximately 22 °C) using a K11 tensiometer (KRÜSS, Hamburg, Germany). Rhamnolipids concentration range was from 10^−3^ to 10^6^ mg/m^3^. Each measurement was conducted in triplicate, and the average was calculated. Afterwards, a surface tension curve versus the decimal logarithm of the biosurfactant concentration (γ = f(logc)) were made, from which the CMC value in water was determined [42]. CMC is the point beyond which a further increase in the biosurfactant concentration does not reduce the surface tension of the water [43].

### 2.3. Alginate-Rhamnolipids Hydrogels Preparation

Ion cross-linking of sodium alginate was performed with the use of aqueous solutions of calcium chloride with various concentrations of 0.05, 0.15, and 0.25 mol/dm^3^. For this purpose, sodium alginate solutions in distilled water (2 g per 0.1 dm^3^ of water) containing selected concentrations of rhamnolipids (above CMC, equal to CMC, below CMC) were prepared. The alginate solutions were poured into the dialysis membrane (VISKING^®^ dialysis tubing made from regenerated cellulose, pore diameter ca. 25 Å, MWCO 12,000–14,000) and placed in the calcium chloride solution for 12, 24, and 36 h. Additionally, control tests were also performed, i.e., alginate hydrogels without the addition of rhamnolipids were prepared and ion-cross-linked with all selected CaCl_2_ concentrations for 12, 24 and 36 h. The resulting ionically cross-linked hydrogels were pulled from the membrane, washed with water, and cut into pieces of equal thickness, which were then subjected to determine their mechanical properties.

### 2.4. Mechanical Properties

The mechanical properties of the hydrogels were characterized using a universal testing machine (Instron model 5543) according to the method described by Hoyer and Bi with co-workers with slight modifications [44,45]. Five cylindrical samples of the test scaffolds (Ø 30 mm × 25 mm) were compressed up to 50% deformation in two cycles at a test speed of 0.5 mm/s. The compression load, flexibility, and cohesiveness were measured.

### 2.5. Experimental Design

The experiments were carried out according to a three-level-three-factor Box–Behnken design (BBD) constructed using Minitab 19 Statistical Software (Minitab Inc., State College, PA, USA). As independent variables, CaCl_2_ concentration (*A*), rhamnolipids concentration (*B*), and ion cross-linking time (*C*) were chosen. All parameters were tested on three levels: low (−1), high (+1), and midpoint (0). The values of levels for individual variables are summarized in Table 1.

The experimental plan implemented in this work is presented in Table 2. For three independent variables (*A*, *B*, *C*), the Box–Behnken design requires 15 experiments. The use of the BBD method allows to adjust the quadratic surface and create the second-order polynomial model presented by Equation (1).

(1) $$y=\beta_{0}+\overset{n}{\underset{i=1}{\sum }}\beta_{i}x_{i}+\overset{n}{\underset{i=1}{\sum }}\beta_{ii}x_{i}^{2}+\overset{n}{\underset{i=1}{\sum }}\;\overset{n}{\underset{j>1}{\sum }}\beta_{ij}x_{i}x_{j}$$

where *y* denotes predicted response, *x* independent variables, *β*_0_ constant term, *β_i_* linear coefficient, *β_ii_* quadratic coefficient, and *β_ij_* interaction coefficient.

## 3. Results and Discussion

### 3.1. CMC of Rhamnolipids in Water

**Figure 1:**
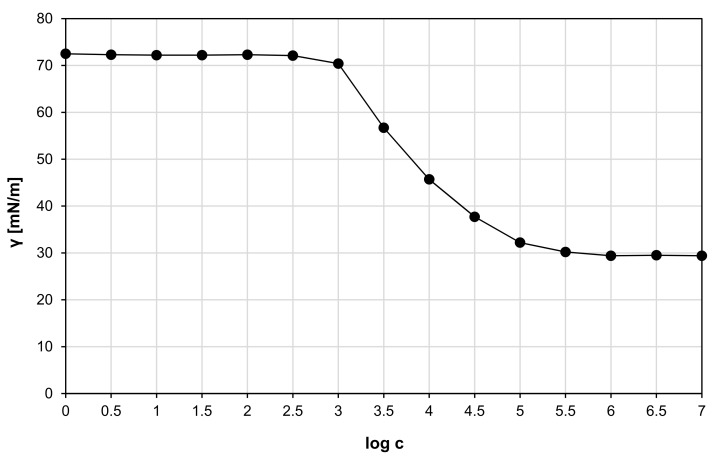
Surface tension function versus the decimal logarithm of the rhamnolipids concentration at room temperature in water.

Surface tension function versus the decimal logarithm of the rhamnolipids concentration is shown in Figure 1. Based on the obtained diagram, the CMC of rhamnolipids in water at room temperature (22 °C) was determined and it was 43.21 mg/dm^3^. This corresponds to the values in the literature, where the CMC of rhamnolipids under the same conditions is reported between 10 and 200 mg/dm^3^, depending on the source of origin [46,47,48,49]. The differences in CMC values occur due to the variation in the length of the lipid chain and the presence of one or two rhamnose residues in rhamnolipids produced by various microorganisms [50].

### 3.2. Alginate Hydrogels without the Addition of Rhamnolipid

In the case of the ionic cross-linking method, divalent Ca^2+^ cations interact with the carboxyl groups of the guluronic residues [6]. The cations diffuse into the sodium alginate solution, creating a gel characterized by a gradient of divalent ions. The degree of cross-linking of the polymer strongly influences the water absorption ability and the mechanical properties of the hydrogel [6]. These properties depend on many factors [51], including the method of cross-linking, the type and concentration of ions used for cross-linking, cross-linking time, additional substances present in the hydrogel and their concentration, the sequence and number of sugar monomers that constitute the alginate, molecular weight of the used polymer, and the concentration of the alginate in solution [52,53,54,55,56].

The mechanical properties of alginate hydrogels were tested by analyzing the texture profile by pressing a cylinder-shaped sample twice. Compressive load was determined as the ratio of the maximum force in the first compression cycle to the compressed surface. Elasticity is a dimensionless quantity expressed as the ratio of the distance of the pin compressing the sample from the beginning of compression to the achievement of the maximum force in the second cycle with the same distance determined in the first cycle. Cohesiveness, also a dimensionless value, was determined as the ratio of the area under the curve in the second compression cycle to the area under the curve in the first compression cycle.

The results of the measurements of the mechanical properties of the control samples are presented in Table 3 (samples C1–C9) and in Supplementary Figure S1. The values of the compressive load of hydrogels without the addition of rhamnolipid indicate that this parameter increases with the increase in the concentration of calcium ions in the cross-linking solution and the time of cross-linking. It was also noticed that the compressive load changes caused by different cross-linking times were smaller at higher concentrations of calcium ions in the hydrogel system. A similar dependence was demonstrated in the measurement of sample cohesiveness. The cohesiveness value depended mainly on the concentration of calcium ions in the cross-linking solution. The cross-linking time for the same sodium chloride concentrations was comparable, which means that the maximum sample cohesiveness was achieved practically after 12 h of alginate cross-linking. The results of measuring the elasticity of alginate hydrogels do not allow to indicate the dependence of this parameter in function of cross-linking time and sodium chloride concentration. Moreover, for the sample cross-linked 12 h in 0.05 mol/dm^3^ sodium chloride solution, it was impossible to determine the elasticity value. This may suggest that for the measurement of sample flexibility, it must meet the following conditions: at least 10 kPa of compression load and 0.30 units of cohesiveness. Siti Fadhilah bt Ibrahim’s team characterized sodium alginate films cross-linked with calcium chloride. The results confirmed that the mechanical properties of the film can be controlled by changing the degree of cross-linking of this biopolymer, resulting from the change in calcium chloride concentration and cross-linking time. An increase in cross-linking time and an increase in calcium chloride concentration increase the mechanical strength of the film [57].

### 3.3. Experimental Design

Table 3 summarizes the results obtained from all 15 experiments planned with Box–Behnken design. The responses are compression load (*y_1_*), flexibility (*y_2_*), and cohesiveness (*y_3_*). Polynomial equations in uncoded units presenting the empirical relationship between the responses and independent variables were determined based on the results from Table 3, and presented in subsequent chapters of this work. Moreover, graphical representations of the designated polynomial equations were prepared in the form of three-dimensional response surfaces as a function of two variables with all parameters maintained at fixed levels. The analysis of these plots allows for a determination whether the relationships between the responses and selected independent variables are linear or quadratic. ANOVA was also performed and Pareto charts were made for all responses, which allowed to indicate the effects of which variables and interactions are statistically significant. A high F-value and *p*-value less than or equal to the significance level (α = 0.05) indicate that the model is statistically significant.

### 3.4. Compression Load

**Figure 2:**
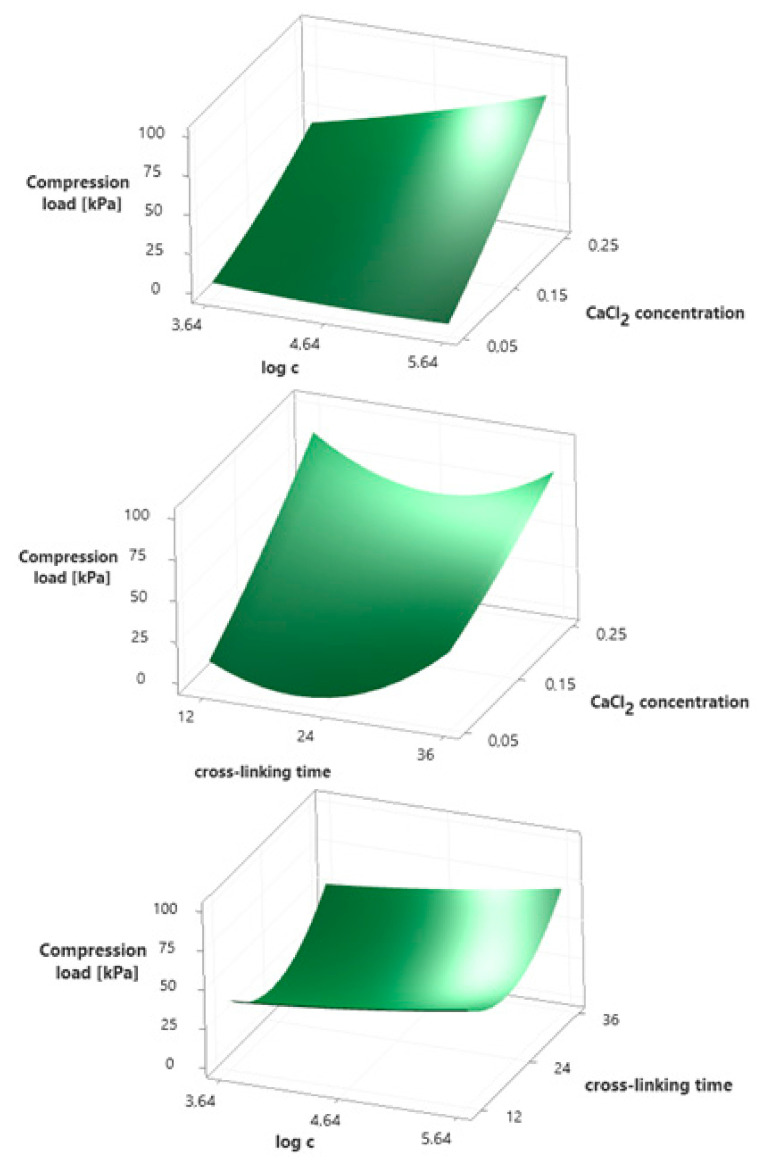
Surface plots presenting the effect of selected independent variables on the compression load of hydrogels.

Three-dimensional response surfaces for the compression load as a function of two variables were prepared and are shown in Figure 2. Polynomial equation presenting the empirical relationship between the compression load (*y*_1_) and independent variables is described by Equation (2). For this model, the coefficient of determination (*R*^2^) equals 97.85%, which indicates a strong agreement between the experimental and predicted responses. According to the Pareto chart (Supplementary Figure S2), the concentration of calcium chloride used for ion cross-linking (*A*) has the greatest impact on the compression load of alginate hydrogels. In addition, a statistically significant effect of the cross-linking time (*C^2^*) and the concentration of rhamnolipids (*B*) present in the hydrogel, as well as the interaction between the CaCl_2_ and rhamnolipids concentrations (*AB*), were observed. This is confirmed by exceeding the baseline (2.57) on the Pareto chart and *p*-values lower than the significance level (*p ≤ α*) included in Supplementary Table S1. Analyzing the response surfaces from Figure 2, it turns out that the relationships of the compression load with the concentration of both CaCl_2_ and rhamnolipids are linear. The higher the concentration of both substances, the higher the compression load. It is also worth noting the interactions between these two independent variables. At the lowest concentration of CaCl_2_, a slight effect of rhamnolipids on the compression load can be seen, while with the increase in calcium chloride concentration, the effect of biosurfactant concentration also increases. Whereas, in the case of cross-linking time, the observed relationship has a quadratic course with the minimum at 24 h. Thus, the alginate hydrogel containing 436.5 mg/dm^3^ (>CMC) of rhamnolipids, cross-linked in 0.25 mol/dm^3^ CaCl_2_ solution for 36 h is characterized by the highest compression load value of 109.5 kPa (calculated using the determined model).

*y_1_ =* 133.4 − 176*A −* 25.4*B* − 7.14*C* + 369*A^2^* + 1.85*B^2^* + 0.1738*C^2^* + 110*AB* − 6.05*AC* + 0.062*BC* (2)

### 3.5. Flexibility

**Figure 3:**
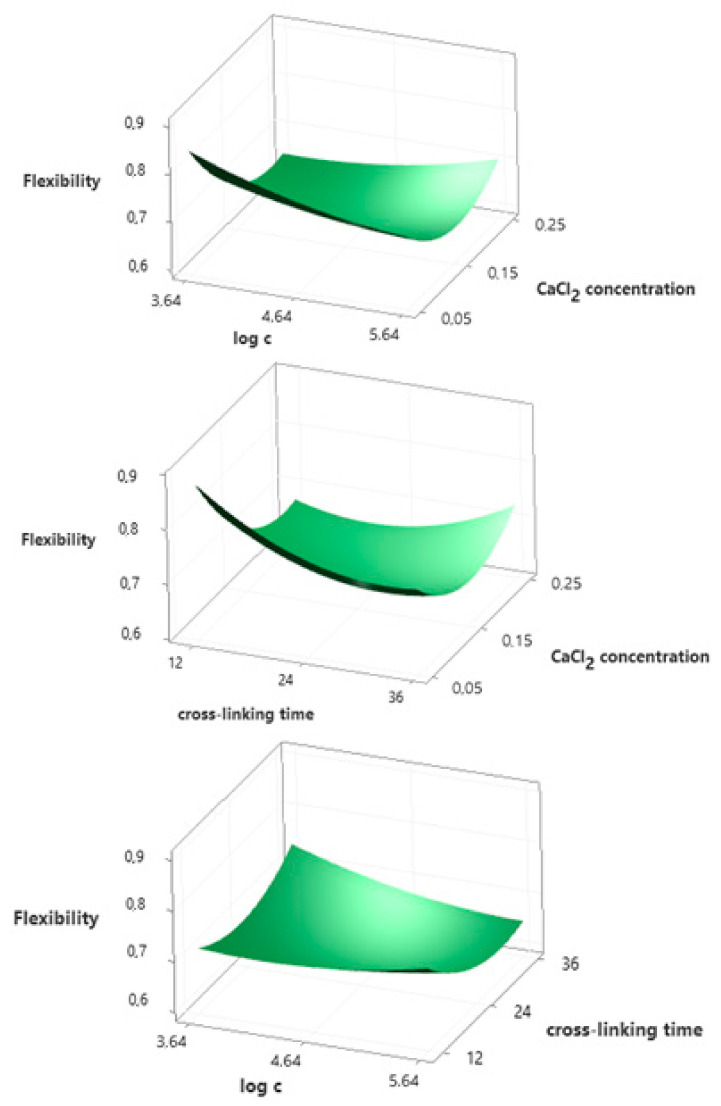
Surface plots presenting the effect of selected independent variables on the flexibility of hydrogels.

ANOVA (Supplementary Table S2) and Pareto chart (Supplementary Figure S3) show that only the concentration of calcium chloride (*A*) has a statistically significant effect on flexibility. The 3D response surface graphs presented in Figure 3 indicate that the relationship between flexibility and CaCl_2_ concentration is square. For the lowest CaCl_2_ concentration tested (0.05 mol/dm^3^), a decrease in flexibility was observed with an increase in the concentration of rhamnolipids. On the other hand, for the highest concentration of 0.25 mol/dm^3^, an increase in the biosurfactant content results in a slight increase in flexibility. The same relationship occurs for the cross-linking time. The empirical relationship between the flexibility (*y_2_*) and independent variables is described by polynomial Equation (3).

*y_2_* = 1.711 − 5.43*A* − 0.187*B* − 0.011*C* + 6.62*A^2^* + 0.0181*B^2^* + 0.000305*C^2^* + 0.422*AB* + 0.038*AC* − 0.00233*BC* (3)

### 3.6. Cohesiveness

**Figure 4:**
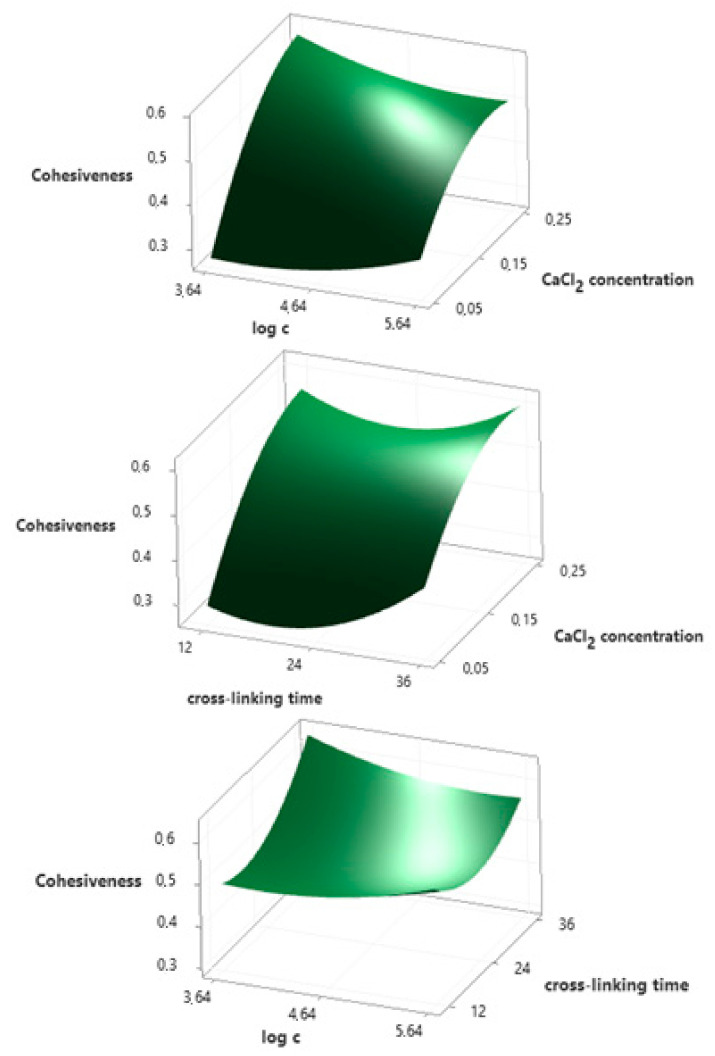
Surface plots presenting the effect of selected independent variables on the cohesiveness of hydrogels.

Polynomial equation presenting the empirical relationship between the cohesiveness (*y_3_*) and independent variables is described by Equation (4). The *R^2^* for this model equals 95.25%, which indicates a strong agreement between the experimental and predicted responses. Response surface plots presented in Figure 4 show that the concentration of calcium chloride (*A*) has the greatest influence on cohesiveness, and the relationship is linear. The ionic cross-linking time (*C^2^*) also has a statistically significant effect on this parameter (quadratic relationship). This is confirmed by the results of the ANOVA (Supplementary Table S3) and the Pareto chart (Supplementary Figure S4). The *p*-value is lower than the significance level (*p ≤ α*) and the exceeding of the baseline (2.571) in the Pareto chart is observed for these two variables.

*y_3_* = 0.281 + 5.2*A* − 0.12*B* − 0.0042*C* − 6.56*A^2^* + 0.0261*B^2^* + 0.000482*C^2^* − 0.365*AB* − 0.0165*AC* − 0.00283*BC* (4)

### 3.7. Reference to Natural Biofilms

The extracellular polymeric substances (EPS) are responsible for biofilm properties, especially the high mechanical resilience of the biofilm, which together render the resident bacteria highly resistant to chemical challenges [58]. In the natural environment the composition of the biofilm matrix is highly heterogeneous and strongly depends on the tested bacterial species, which makes it difficult to predict biofilm properties. Therefore, in our study, the alginate hydrogel was used as a model biofilm.

The obtained results show that the presence of the biosurfactant has the greatest impact on the compression load among the examined mechanical parameters. Kaygusuz and co-workers proposed mechanisms related to cross-linking of sodium alginate with calcium ions in the presence of anionic detergent, sodium dodecyl sulfate (SDS), and nonionic detergent Brij 35, changing the mechanical properties of alginate hydrogels [40]. They pointed out that the addition of SDS may increase the negative charge density and therefore a higher amount of calcium ions localized around the alginate chain. This increased amount of crosslinkers causes an increase in the Young modulus and stiffness of the hydrogel. Similar to SDS, rhamnolipids are the anionic biosurfactants, but its acidic character results from the presence of carboxylic groups in their molecules. They have a relatively large hydrophilic group composed of rhamnose. Comparing the compression values for alginate gels without biosurfactants and obtained in their presence, it can be seen that adding rhamnolipid molecules in the concentration range to the CMC value lowers the compression load. Only when the biosurfactant is added in an amount exceeding the concentration of CMC, the compression values for alginate gels are higher. However, in the case of alginate gels with SDS, no studies were conducted in the range below the CMC value. In biofilms, the presence of rhamnolipid molecules does not exceed the CMC value (according to [59], the highest concentration of rhamnolipids in a biofilm is about 100 µM), therefore it may be confirmed by the influence of this biosurfactant on the formation of the biofilm structure [59]. Moreover, rhamnolipids interact with the hydrophobic part of the alginate copolymer chains, and then the hydrophilic groups of adsorbed biosurfactant molecules create additional calcium ion trapping sites. Therefore, for higher surfactant concentrations, it is possible to introduce more calcium ions cross-linking the alginate molecules.

The obtained research results may contribute to the understanding of the high mechanical resistance of biofilms formed by bacteria producing rhamnolipids. An example of such microorganisms is the opportunistic pathogen *Pseudomonas aeruginosa*. Extensive research is carried out to understand the properties of biofilms created by these microorganisms [32,60,61], and some of the conducted studies have shown that the viscoelastic properties of these biofilms are resistant to chemical treatment and strong shear forces, allowing to efficiently recover from mechanical damage [62]. So far, the function of rhamnolipids in biofilms, consisting in preserve the pores and channels between microcolonies, enabling the passage of liquid and nutrients within mature biofilms, has been primarily emphasized [61]. However, the results of our research indicate that their influence on the mechanical properties of biofilms is also likely.

## 4. Conclusions

In summary, the presence of rhamnolipids changes the mechanical properties of the alginate hydrogel. The addition of this biosurfactant primarily affects the compression load, and the value of this parameter is lower for hydrogels produced with rhamnolipid concentration below CMC than for hydrogels obtained in pure water. On the other hand, the addition of biosurfactants in an amount exceeding CMC causes an increase in the compression load. Measurements of flexibility and cohesiveness showed no significant differences between hydrogels prepared without and with rhamnolipids in the tested concentration range. This study adds to the increasing knowledge of the influence of rhamnolipids on the mechanical properties of biofilms. A more standardized approach, together with the evaluation of a wider range of environmental signals, may allow us to control biofilm formation in the future, facilitate biofilm removal, and possibly generate biofilms with specific properties for industrial applications.

## Figures and Tables

**Table 1:** Range and levels of parameters in Box-Behnken experimental design.

Factors	Parameters	Coded Levels		
		−1	0	1
*A*	CaCl_2_ concentration [mol/dm^3^]	0.05	0.15	0.25
*B*	Rhamnolipids concentration [log c]	3.64	4.64	5.64
*C*	Ion cross-linking time [h]	12	24	36

**Table 2:** Box-Behnken matrix.

Run	*A*	*B*	*C*	CaCl_2_ Concentration [mol/dm^3^]	Rhamnolipids Concentration [log c]	Ion Cross-Linking Time [h]
1	−1	−1	0	0.05	3.64	24
2	+1	−1	0	0.25	3.64	24
3	−1	+1	0	0.05	5.64	24
4	+1	+1	0	0.25	5.64	24
5	−1	0	−1	0.05	4.64	12
6	+1	0	−1	0.25	4.64	12
7	−1	0	+1	0.05	4.64	36
8	+1	0	+1	0.25	4.64	36
9	0	−1	−1	0.15	3.64	12
10	0	+1	−1	0.15	5.64	12
11	0	−1	+1	0.15	3.64	36
12	0	+1	+1	0.15	5.64	36
13	0	0	0	0.15	4.64	24
14	0	0	0	0.15	4.64	24
15	0	0	0	0.15	4.64	24

**Table 3:** Mechanical properties of the hydrogels prepared according to the Box-Behnken plan (samples 1–15), and hydrogels without rhamnolipids (control samples C1–C9).

Run	CaCl_2_ Concentration [mol/dm^3^]	Rhamnolipids Concentration [log c]	Ion Cross-linking Time [h]	Compression Load [kPa]	Flexibility [−]	Cohesiveness [−]
1	0.05	3.64	24	5.74 ± 0.06	0.861 ± 0.009	0.301 ± 0.007
2	0.25	3.64	24	35.18 ± 0.66	0.673 ± 0.006	0.613 ± 0.010
3	0.05	5.64	24	9.60 ± 0.59	0.707 ± 0.005	0.311 ± 0.009
4	0.25	5.64	24	83.04 ± 3.93	0.688 ± 0.004	0.477 ± 0.012
5	0.05	4.64	12	6.38 ± 0.48	-	0.294 ± 0.008
6	0.25	4.64	12	89.41 ± 2.48	0.662 ± 0.010	0.552 ± 0.005
7	0.05	4.64	36	38.25 ± 2.65	0.763 ± 0.009	0.425 ± 0.011
8	0.25	4.64	36	95.24 ± 0.38	0.728 ± 0.011	0.604 ± 0.013
9	0.15	3.64	12	43.50 ± 1.97	0.695 ± 0.005	0.463 ± 0.015
10	0.15	5.64	12	55.32 ± 2.04	0.773 ± 0.014	0.591 ± 0.006
11	0.15	3.64	36	52.65 ± 1.56	0.703 ± 0.008	0.598 ± 0.005
12	0.15	5.64	36	67.45 ± 2.52	0.669 ± 0.007	0.590 ± 0.010
13	0.15	4.64	24	27.85	0.648	0.465
14	0.15	4.64	24	26.56	0.638	0.477
15	0.15	4.64	24	29.14	0.658	0.453
C1	0.05	-	12	7.50 ± 0.33	-	0.349 ± 0.005
C2	0.05	-	24	22.76 ± 1.01	0.907 ± 0.005	0.312 ± 0.004
C3	0.05	-	36	38.13 ± 1.15	0.709 ± 0.007	0.406 ± 0.005
C4	0.15	-	12	66.94 ± 3.47	0.744 ± 0.008	0.509 ± 0.008
C5	0.15	-	24	68.88 ± 2.43	0.688 ± 0.010	0.535 ± 0.010
C6	0.15	-	36	71.20 ± 4.02	0.751 ± 0.004	0.479 ± 0.007
C7	0.25	-	12	82.48 ± 4.87	0.673 ± 0.004	0.587 ± 0.011
C8	0.25	-	24	91.72 ± 1.40	0.738 ± 0.012	0.585 ± 0.009
C9	0.25	-	36	93.14 ± 3.60	0.824 ± 0.006	0.558 ± 0.013

## Data Availability

The data presented in this study are openly available in MOST Wiedzy Open Research Data Catalog at doi:10.34808/5k76-8655.

## Supplementary Materials

The following are available online at https://www.mdpi.com/article/10.3390/ijms22136840/s1.
