# Supplementary material for: Influence of Rhamnolipids and Ionic Cross-Linking Conditions on the Mechanical Properties of Alginate Hydrogels as a Model Bacterial Biofilm
Source: Int J Mol Sci. 2021 Jun 25;22(13):6840. doi: 10.3390/ijms22136840 (PMC8269414; doi:10.3390/ijms22136840)
Supplement: Supplementary file 1 [file ijms-22-06840-s001.zip › ijms-1243967-supplementary.pdf]

## Supplementary Materials

# Influence of rhamnolipids and ionic cross-linking conditions on the mechanical properties of alginate hydrogels as a model bacterial biofilm

Czaplicka Natalia [1*], Mania Szymon [2], Konopacka-Łyskawa Donata [1]

[1] Department of Process Engineering and Chemical Technology, Faculty of Chemistry, Gdańsk University of Technology, Narutowicza 11/12, 80-233 Gdańsk, Poland

[2] Department of Chemistry, Technology and Biotechnology of Food, Faculty of Chemistry, Gdańsk University of Technology, Narutowicza 11/12, 80-233 Gdańsk, Poland

* corresponding author: natalia.czaplicka@pg.edu.pl, tel. +48 58 347 21 14

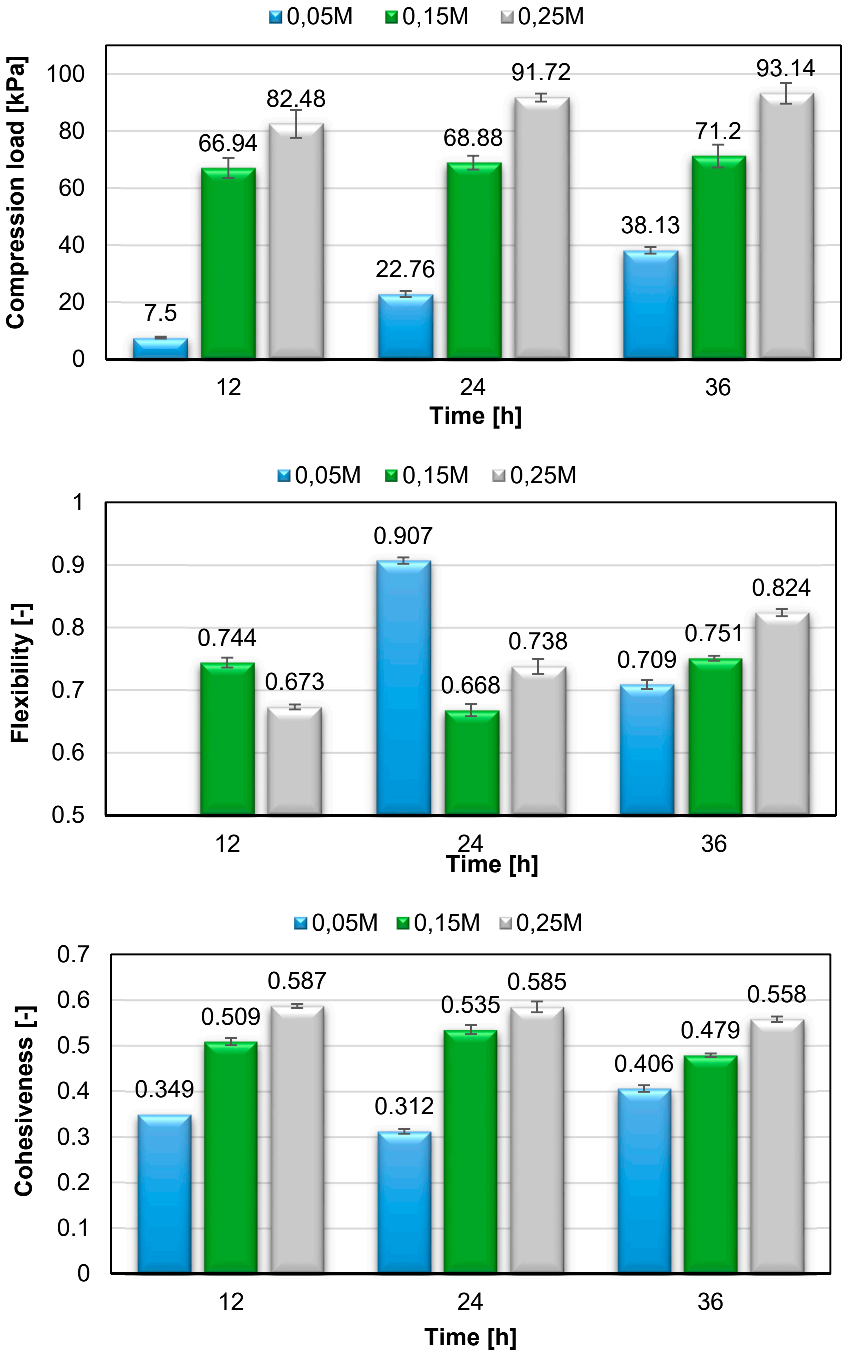


**Figure S1.** Mechanical properties of the control hydrogels samples as a function of cross-linking time and $CaCl_2$ concentration.

**Table S1.** ANOVA results of the quadratic model for compression load

| Source | Degrees of freedom (df) | Sum of squares (SS) | Mean square (MS) | F-value | p-value |
|---|---|---|---|---|---|
| Regression | 9 | 11374.9 | 1263.87 | **25.25** | **0.001** |
| Linear | 3 | 8352.9 | 2784.29 | **55.62** | **0.000** |
| A – $CaCl_2$ concentration | 1 | 7194.0 | 7194.00 | **143.72** | **0.000** |
| B – rhamnolipids concentration | 1 | 767.1 | 767.14 | **15.33** | **0.011** |
| C – ion cross-linking time | 1 | 391.7 | 391.72 | **7.83** | **0.038** |
| Square | 3 | 2325.0 | 774.98 | **15.48** | **0.006** |
| $A^2$ | 1 | 50.3 | 50.27 | 1.00 | 0.362 |
| $B^2$ | 1 | 12.6 | 12.64 | 0.25 | 0.637 |
| $C^2$ | 1 | 2313.2 | 2313.23 | **46.21** | **0.001** |
| 2-way interaction | 3 | 697.1 | 232.35 | 4.64 | 0.066 |
| AB | 1 | 484.0 | 484.00 | **9.67** | **0.027** |
| AC | 1 | 210.8 | 210.83 | 4.21 | 0.095 |
| BC | 1 | 2.2 | 2.22 | 0.04 | 0.842 |
| Residual error | 5 | 250.3 | 50.06 | - | - |
| Lack of fit | 3 | 247.0 | 82.32 | **49.47** | **0.020** |
| $R^2$ | | **0.9785** | | | |

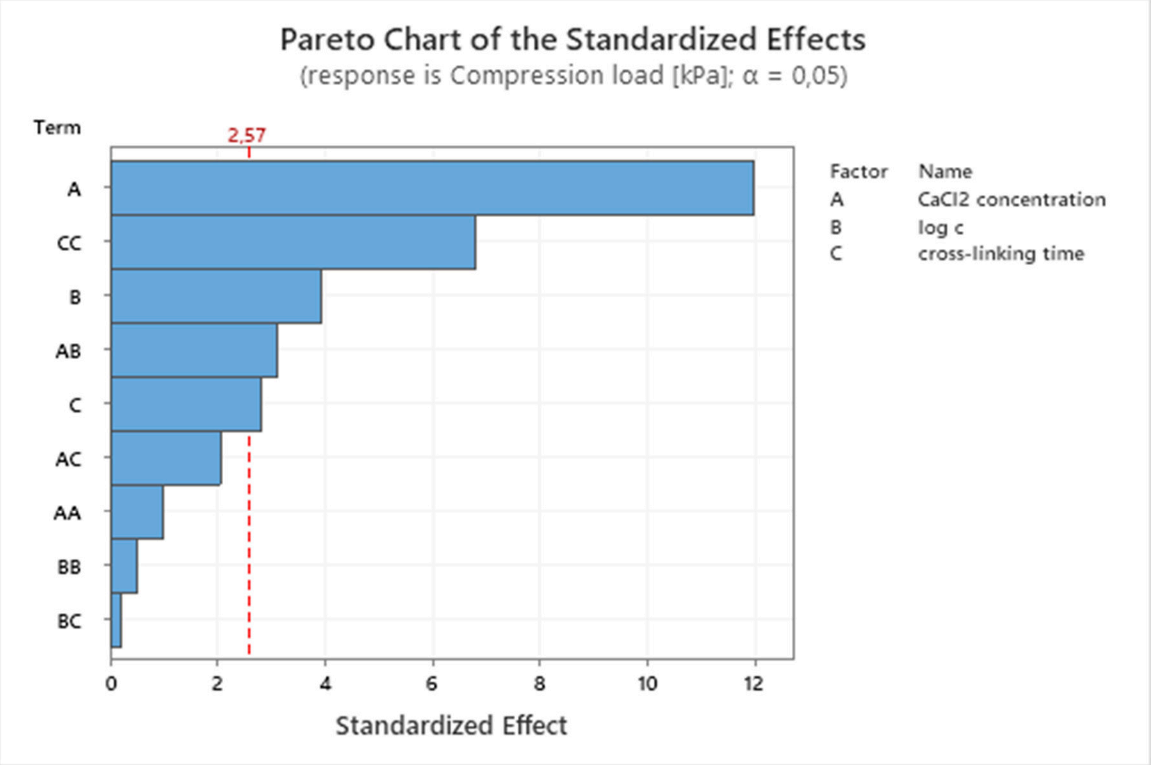


**Figure S2.** Pareto chart of the standardized effects for compression load.

**Table S2.** ANOVA results of the quadratic model for flexibility

| Source | Degrees of freedom (df) | Sum of squares (SS) | Mean square (MS) | F-value | p-value |
|---|---|---|---|---|---|
| Regression | 9 | 0.042461 | 0.004718 | 3.85 | 0.104 |
| Linear | 3 | 0.018727 | 0.006242 | 5.09 | 0.075 |
| A – $CaCl_2$ concentration | 1 | 0.017595 | 0.017595 | **14.35** | **0.019** |
| B – rhamnolipids concentration | 1 | 0.001128 | 0.001128 | 0.92 | 0.392 |
| C – ion cross-linking time | 1 | 0.001789 | 0.001789 | 1.46 | 0.294 |
| Square | 3 | 0.018144 | 0.006048 | 4.93 | 0.079 |
| $A^2$ | 1 | 0.013142 | 0.013142 | **10.72** | **0.031** |
| $B^2$ | 1 | 0.000979 | 0.000979 | 0.80 | 0.422 |
| $C^2$ | 1 | 0.005792 | 0.005792 | 4.72 | 0.095 |
| 2-way interaction | 3 | 0.014440 | 0.004813 | 3.93 | 0.110 |
| AB | 1 | 0.007140 | 0.007140 | 5.82 | 0.073 |
| AC | 1 | 0.004163 | 0.004163 | 3.40 | 0.139 |
| BC | 1 | 0.003136 | 0.003136 | 2.56 | 0.185 |
| Residual error | 4 | 0.004904 | 0.001226 | - | - |
| Lack of fit | 2 | 0.004704 | 0.002352 | **23.52** | **0.041** |
| $R^2$ | | **0.8965** | | | |

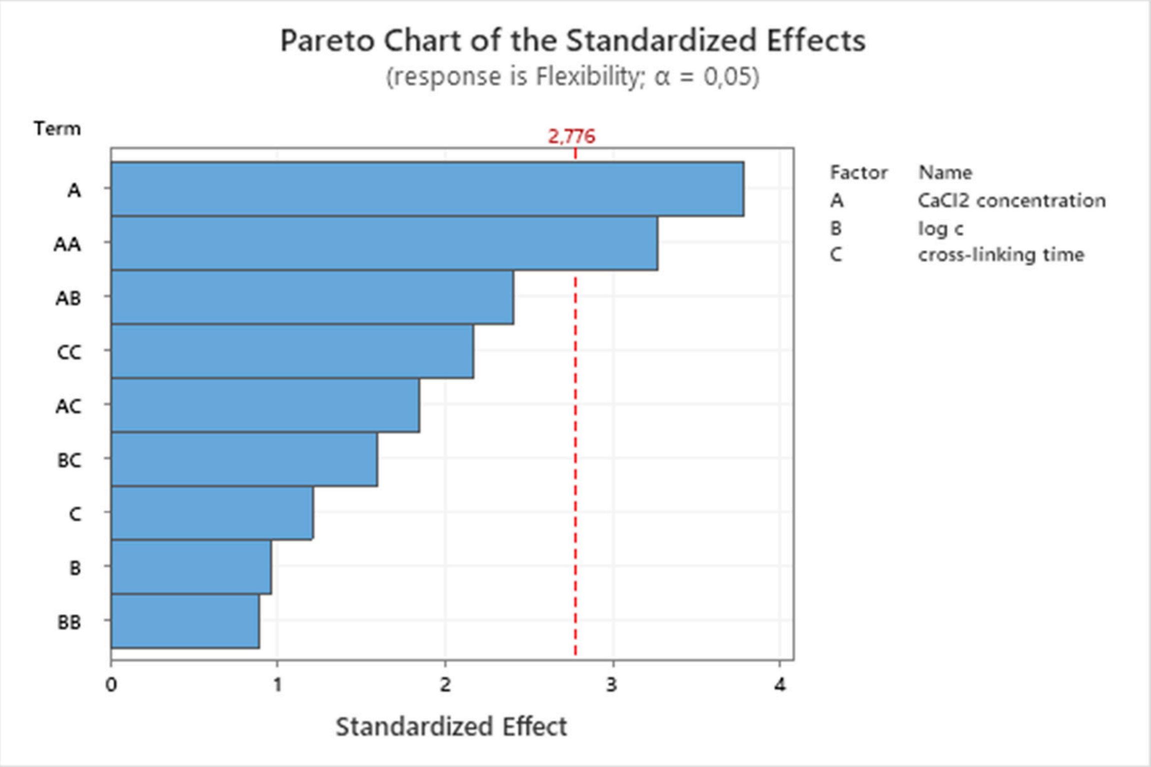


**Figure S3.** Pareto chart of the standardized effects for flexibility.

**Table S3.** ANOVA results of the quadratic model for cohesiveness

| Source | Degrees of freedom (df) | Sum of squares (SS) | Mean square (MS) | F-value | p-value |
|---|---|---|---|---|---|
| Regression | 9 | 0.167702 | 0.018634 | **11.14** | **0.008** |
| Linear | 3 | 0.117219 | 0.039073 | **23.36** | **0.002** |
| A – $CaCl_2$ concentration | 1 | 0.104653 | 0.104653 | **62.57** | **0.001** |
| B – rhamnolipids concentration | 1 | 0.000004 | 0.000004 | 0.00 | 0.961 |
| C – ion cross-linking time | 1 | 0.012561 | 0.012561 | 7.51 | 0.041 |
| Square | 3 | 0.038970 | 0.012990 | **7.77** | **0.025** |
| $A^2$ | 1 | 0.015901 | 0.015901 | **9.51** | **0.027** |
| $B^2$ | 1 | 0.002520 | 0.002520 | 1.51 | 0.274 |
| $C^2$ | 1 | 0.017771 | 0.017771 | **10.62** | **0.022** |
| 2-way interaction | 3 | 0.011513 | 0.003838 | 2.29 | 0.195 |
| AB | 1 | 0.005329 | 0.005329 | 3.19 | 0.134 |
| AC | 1 | 0.001560 | 0.001560 | 0.93 | 0.378 |
| BC | 1 | 0.004624 | 0.004624 | 2.76 | 0.157 |
| Residual error | 5 | 0.008363 | 0.001673 | - | - |
| Lack of fit | 3 | 0.008075 | 0.002692 | 18.69 | 0.051 |
| $R^2$ | | **0.9525** | | | |

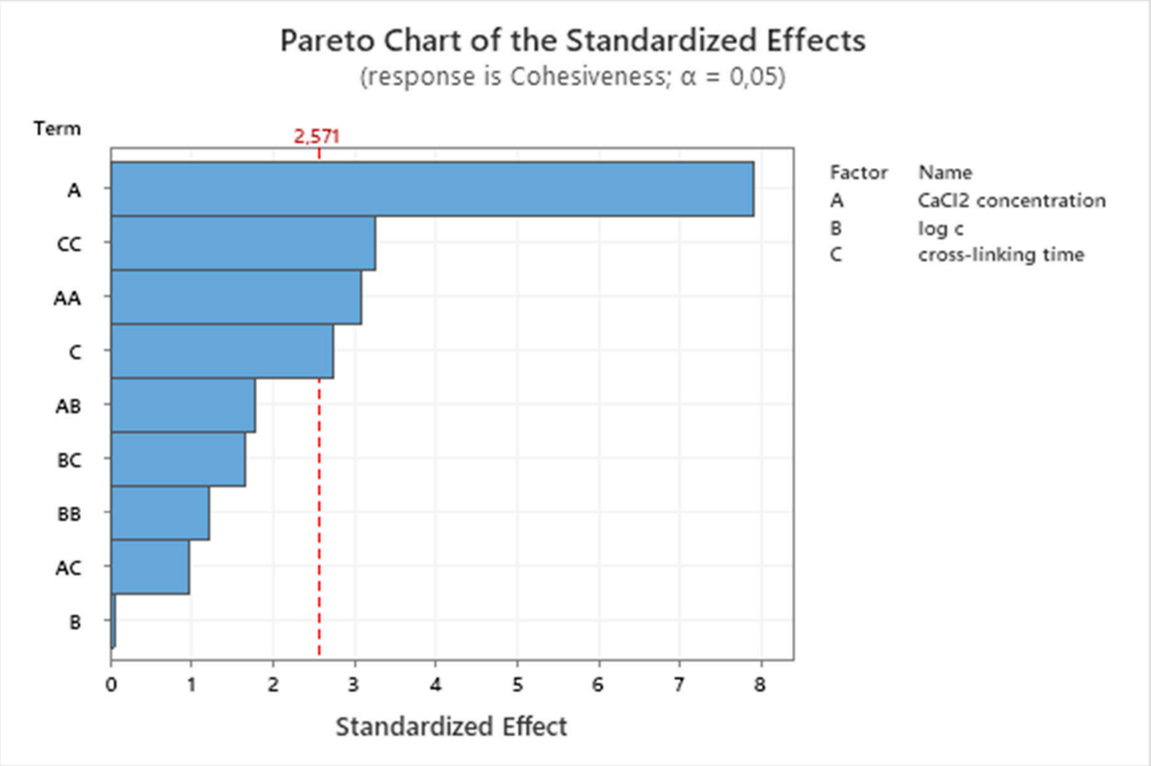


**Figure S4.** Pareto chart of the standardized effects for cohesiveness.
